# Supplementary material for: Thrombotic Long-Term Consequences of SARS-CoV-2 Infection in Patients with Compensated Cirrhosis: A Propensity Score-Matched Analysis of a U.S. Database
Source: Diseases. 2024 Jul 17;12(7):161. doi: 10.3390/diseases12070161 (PMC11276382; doi:10.3390/diseases12070161)
Supplement: Supplementary file 1 [file diseases-12-00161-s001.zip › diseases-3068280-supplementary.pdf]

Table S1. ICD-10 codes used in inclusion and exclusion criteria

Inclusion Criteria	Code	Exclusion Criteria	Code
Age (at least 18 years)	Age	Presence of prosthetic heart valve	Z95.2
Alcoholic cirrhosis of liver without ascites	K70.30	Atrial fibrillation and flutter	I48
Hepatic fibrosis	K74.0	Acute embolism and thrombosis of unspecified deep veins of lower extremity	I82.40
Alcoholic cirrhosis of liver	K70.3	Pulmonary embolism	I26
Primary biliary cirrhosis	K74.3	Esophageal varices without bleeding	I85.00
Other cirrhosis of liver	K74.69	Hepatic failure unspecified with coma	K72.91
Unspecified cirrhosis of liver	K74.60	Hepatic encephalopathy	K76.82
Fibrosis and cirrhosis of liver	K74	Esophageal varices with bleeding	I85.01
Secondary biliary cirrhosis	K74.4	Spontaneous bacterial peritonitis	K65.2
Biliary cirrhosis unspecified	K74.5	Secondary esophageal varices	I85.1
Alcoholic fibrosis and sclerosis of liver	K70.2	Esophageal varices	I85.0
SARS coronavirus 2 IgG IgM Ab [Presence] in Serum or Plasma		Esophageal varices	I85
SARS coronavirus 2 and related RNA [Presence]		Unspecified jaundice	R17
		Ascites	R18
		Hepatorenal syndrome	K76.7
		Other ascites	R18.8
		Alcoholic cirrhosis of liver with ascites	K70.31

Table S2. Codes used in PSM components.

Propensity Score Matching Component	Code
**Age at Index**	AI
**White**	2106-3
**Female**	F
**Not Hispanic or Latino**	2186-5
**Hispanic or Latino**	2135-2
**Black or African American**	2054-5
**Male**	M
**Asian**	2028-9
**Atherosclerotic heart disease of native coronary artery**	I25.1
**Chronic kidney disease (CKD)**	N18
**Other chronic obstructive pulmonary disease**	J44
**Essential (primary) hypertension**	I10
**Diabetes mellitus**	E08-E13
**Pulmonary embolism**	I26
**Cerebral infarction**	I63
**PLATELET AGGREGATION INHIBITORS**	BL117
**ANTICOAGULANTS**	BL110
**Warfarin**	11289
